# Synergy between immune system and antibiotics drives infection control in mice

**DOI:** 10.3389/fimmu.2025.1719808

**Published:** 2026-01-21

**Authors:** Rajalekshmy G. Padmakumari, Ruchi Roy, Foyez Mahmud, Deepa Dehari, Getnet Tesfaw, Christi Thomas, Athena M. Soulika, Roslyn Rivkah Isseroff, Sasha H. Shafikhani

**Affiliations:** 1 Department of Dermatology, University of California Davis, Sacramento, CA, United States; 2 UICentre for Drug Discovery, College of Pharmacy, University of Illinois at Chicago, Chicago, IL, United States; 3 VeriSim Life Inc., San Francisco, CA, United States; 4 Department of Dermatology, Shriners Hospital for Children, Sacramento, CA, United States; 5 Dermatology Section, VA Northern California Health Care System, Mather, CA, United States; 6 Microbiology Graduate Group (MGG), University of California Davis, Sacramento, CA, United States; 7 Graduate Group in Immunology (GGI), University of California Davis, Sacramento, CA, United States; 8 Cancer Center, University of California Davis, Sacramento, CA, United States

**Keywords:** antibiotics, immune system, immunocompetent host, immunocompromised host, immunomodulator, infection, *Pseudomonas aeruginosa*, leukocyte

## Abstract

**Background:**

Antibiotics and host immunity are traditionally viewed as independent defenses, with antibiotics reducing bacterial load to levels manageable by the immune system. Modeling studies, however, predict that synergy between these defenses is critical for infection control, but this has not been experimentally verified.

**Methods:**

We tested this concept using a *Pseudomonas aeruginosa* wound infection model in immunocompetent (C57BL/6) and immunocompromised (NSG) mice treated with systemic tobramycin.

**Results:**

In C57BL/6 mice, tobramycin-mediated bacterial killing increased pathogen-associated molecular patterns (PAMPs) - namely lipopolysaccharide (LPS) - which in turn amplified local inflammation, enhancing antibiotic efficacy in a manner largely dependent on neutrophils. In contrast, NSG mice failed to potentiate tobramycin bacterial killing to increase PAMPs and mount Tobramycin-induced boost in immune activation, resulting in reduced infection control. Importantly, topical PAMPs (LPS and N-formyl-methionyl-leucyl-phenylalanine (fMLP)) restored immune activation and improved infection control in NSG mice in a manner that was also dependent on neutrophil’s function.

**Conclusion:**

These findings provide direct experimental evidence that antibiotic efficacy requires synergy with host immunity. They highlight the therapeutic potential of augmenting innate immune activation to improve infection outcomes, particularly in immunocompromised patients.

## Introduction

Antibiotics and the immune system have traditionally been viewed as distinct and independent arms of defenses against infection, with antibiotics thought to lower the bacterial burden to levels manageable by the immune system (1, 2). However, it is widely recognized that systemic antibiotics are markedly less effective in immunocompromised patients, even when the infecting organism is sensitive to the administered antibiotic (3–8). This reduced effectiveness has often been attributed to impairments such as therapy-induced neutropenia (as seen in chemotherapy or bone marrow-ablated transplant patients) (3, 9–11), or dysregulated innate immunity and neutrophil dysfunction in conditions like diabetes (6, 12). Recent mathematical models have indicated that synergy between the antibiotics and immune system is essential for infection control *in vivo* but such synergy, particularly under immunocompromised conditions, has not been experimentally validated (13).

In this study, we address this critical knowledge gap by demonstrating a direct synergy between a systemic antibiotic and host immunity in reducing infection *in vivo*. Using a murine wound infection model with *Pseudomonas aeruginosa*, we show that in immunocompetent C57BL/6 mice, initial bacterial killing by tobramycin results in increased bioactive bacterial components (i.e., LPS), which activate toll-like receptors (TLRs) and initiate a local inflammatory cascade, leading to immune cell recruitment and functional synergy between antibiotic treatment and immune system. In contrast, imunodeficient NSG mice fail to mount this secondary immune activation, due to reduced bioactive pathogen-associated molecular patterns (PAMPs), resulting in reduced bacterial clearance. Notably, topical administration of PAMPs in NSG mice enhanced this secondary immune activation and improved infection outcomes by enhancing neutrophil activity. These findings provide direct experimental evidence that immune activation is not only complementary but necessary for optimal antibiotic efficacy. Our findings further underscore the therapeutic potential of engaging innate immune system to augment infection control, particularly in patients with compromised immune function.

## Materials and methods

The list of all the reagents, antibodies, primers, and their sources can be found in the **Supplementary Table 1**.

### Procedures related to animal studies

We have an approval from the Institutional Animal Care and Use Committee (IACUC No: 24062) to conduct research as indicated. All procedures complied strictly with the standards for care and use of animal subjects as stated in the Guide for the Care and Use of Laboratory Animals (Institute of Laboratory Animal Resources, National Academy of Sciences, Bethesda, MD, USA). We obtained 8-weeks old C57BL/6 and NOD.Cg-*Prkdc^scid^ Il2rgtm1Wjl/*SzJ (NSG) immunocompromised mice and C57BL/6 immunocompetent mice from Jackson Laboratories (Bar Harbor, ME). These Mice were allowed to acclimate to the environment for 1 week prior to experimentation. Wounding and wound infection were carried out as we described previously (14–16). Briefly, the full-thickness excisional wounding was performed by sterile biopsy punches (5-mm diameter, AcudermH, Lauderdale, FL) in anesthetized mice. Analgesics were included in the procedure as per the IACUC protocol. Buprenorphine injection was given 0.05-0.1 mg/kg, Subcutaneous (SC) 30 minutes before wounding and every 8–12 hours post-surgery for 48 hours. Each mouse received four equidistanced wounds on the back below the shoulder blades. Tobramycin was administered by intraperitoneal injection (i.p.) at 0.35 mg/mL in 0.2 mL sterile saline (3.5 mg/kg), 1 hour prior to wounding, following previously described dosing protocols and procedures (17–19). We selected tobramycin because it is among the most potent bactericidal aminoglycosides active against even gentamycin-resistant *P. aeruginosa* (20). We also used the systemic route of antibiotic administration, as this is the recommended route in clinical guidelines for antimicrobial therapy in neutropenic, immunocompromised patients (3). In addition, we administered tobramycin 1 hour prior to infection because delivering a systemic antibiotic within 60 minutes before surgical incision is the standard for antibiotic prophylaxis guideline, and wound surgery falls under this recommendation (2). Finally, dosing of tobramycin was determined from previous publications, based on its efficacy in controlling infection without causing adverse effects, such as nephrotoxicity or ototoxicity (18, 21–23). The number of mice per group was determined by an *a priori* power analysis based on our primary outcome measure (infection burden quantified by CFU), using previously published datasets from our laboratory (15, 24–26), with an estimated effect size of 1.4 (Cohen’s d), assuming a two-tailed test, α = 0.05, and 80% power.

### Bacteria infection

We used *Pseudomonas aeruginosa* (PA103) bacterial strain for infection in this study. This strain has been described previously (19, 27, 28). Bacteria were resuspended in 10 µl sterile PBS and added topically to the wounds at 1x 10^6^ colony forming unit (CFU)/wound. Infection levels in wounds were evaluated by determining the number of bacterial CFU and normalized per gram of wound tissues, as described (15, 24–26). Lipopolysaccharides (LPS) and fMLP were added respectively at 100 ng or 50 ng per wound, prior to infection. LPS and fMLP doses were chosen based on their demonstrated effectiveness to reduce wound infections in mice (19, 29).

### Histopathological evaluation

Leukocytes infiltration into the wound bed was performed by H&E staining as we described previously (15, 30). Activated neutrophil levels in wounds was assessed by wound tissues’ myeloperoxidase (MPO) contents by ELISA as we described (19, 30, 31). The histological data were normalized per field of view.

### Western blot analyses

We performed Western immunoblotting on tissue lysates, using the indicated antibodies after normalization to GAPDH loading control as described (25, 27, 31–34).

### Bioactive LPS measurements in wound

Bioactive LPS in wound was determined by serial dilution using HEK-Blue LPS detection kit 2 from InvivoGen following the manufacturer’s protocol.

### Reagents & antibodies (for western blotting)

The list of reagents, mice, primers for RT-PCR, and antibodies and their sources are included in the **Supplementary Table 1**.

### Gene transcription analysis

Gene expression at mRNA level was assessed by real-time polymerase chain reaction (RT-PCR), using gene-specific primer pairs (included in **Supplementary Table 1**) by the Applied Biosystems QuantStudio™ 7 Flex Real-Time PCR System as described (16, 35–37). The data were calculated using the 2^−ΔΔCt^ method and normalized to 18S.

### Neutrophil depletion

Neutrophils were depleted in mice as described previously (30). Briefly, 400µg anti-Ly6G antibody or IgG2a isotype (control), were injected intraperitoneally in mice a week before wounding and infection. Additional two consecutive doses of antibodies were administered with 100µg at 36 and 12 hours prior to wounding and infection experiment. Neutrophil depletion was confirmed by flow cytometry analysis (FACS). FACS analysis of PBMC was performed using fluorescence conjugated anti-CD45 (clone 30F11, # 103128), anti-CD11b (clone M1/70, #101251), anti-Ly6G (clone 1A8, 127616) and Zombie NIR_TM_ Fixable Viability Kit (#423105). Compensation control was achieved with appropriate counting beads according to the manufacturer’s instructions and the acquisitions were performed by Attune NxT cytometer (Thermo Fisher Scientific) equipped with four lasers and 16-parameter configuration.

### Statistical analysis

Statistical analyses between groups were conducted by One-way ANOVA with additional *post hoc* testing, and pair-wise comparisons between groups were performed or by unpaired Student’s *t*-test. Data are presented as Mean ± SEM. *P*-values less than or equal to 0.05 were considered as significant. Of note, we evaluated normality and variance homogeneity for all datasets included in parametric analyses before proceeding to student’s *t* test and one way ANOVA by using GraphPad prism (version 10.4.2). Normality was assessed using the Shapiro–Wilk test (n < 30), and homogeneity of variance was evaluated using F-test (for 2 groups) and Brown-Forsythe test (more than two groups). In all cases, the data met these assumptions. These data are available in **Supplementary Figures S5** and **Supplementary Figures S6**.

### Schematic diagram

Biorender software was used to generate the schematic diagram in **Figure 6**.

## Results

### Tobramycin treatment boosts immunity against *P. aeruginosa* infection in immunocompetent C57BL mice

We used a full-thickness excisional wound infection model (14, 15) to investigate the potential synergy between host immunity and systemic antibiotic treatment during infection. Immunocompetent C57BL/6 mice received intraperitoneal (i.p.) injections of either PBS (control) or tobramycin (3.5 mg/kg), as described in Materials and Methods, 1 hour before infection with PA103 (10^6^ CFU/wound), a clinical *Pseudomonas aeruginosa* isolate shown to cause infection and tissue damage in wound (14, 30). Tobramycin dosing was based on prior studies (18, 19).

**Figure 1:**
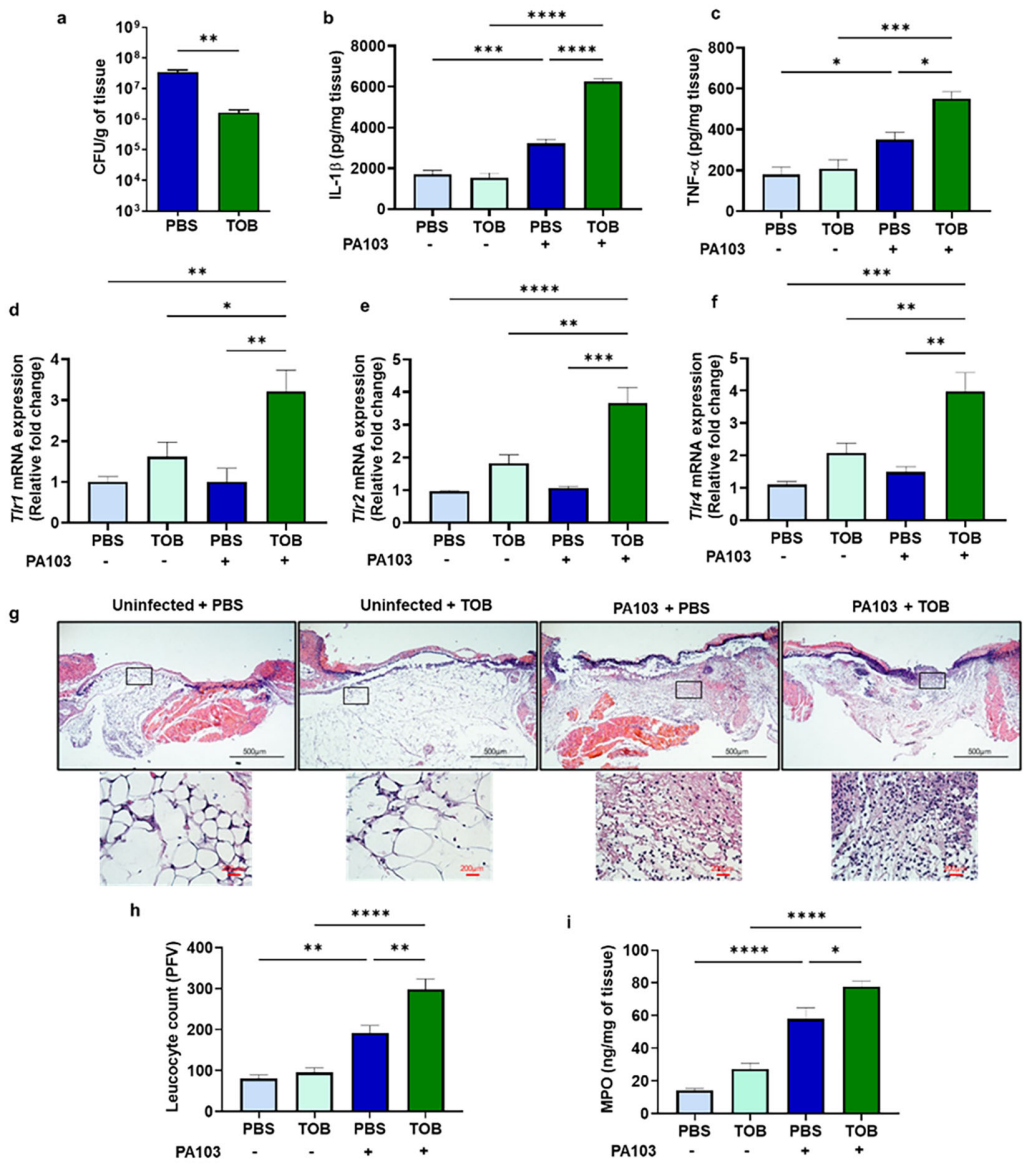
Tobramycin treatment boosts immunity against *P. aeruginosa* infection in C57BL wounds. Normal C57B mice were injected intraperitoneally (i.p.) with PBS (mock) and Tobramycin (3.5 mg/kg) 1 hour prior to wounding. Wounds were infected with PA103 (10^6^ CFU/wound). Wound tissues were harvested on day 1 post-infection and assessed for; **(a)** their bacterial contents by CFU analysis; **(b, c)** for their IL-1β and TNF-α proinflammatory cytokines by ELISA; **(d-f)** for the mRNA expression analysis of Tlr1, Tlr2, and Tlr4 by RT-PCR (normalized to 18S); **(g, h)** for their proinflammatory leukocytes contents using histological analysis using H&E staining; **(i)** and for their activated neutrophils contents by MPO ELISA. Black scale bars = 500µm and red scale bars = 200µm. Corresponding data were plotted as the Mean ± SEM. Statistical comparison between groups was determined using one-way ANOVA with Tukey’s *post hoc* test (N = 4 mice/group; ns, not significant, *p<0.05; **p<0.01; ***p<0.001; ****p<0.0001).

We collected wound tissues 24 hours after infection and antibiotic treatment and analyzed them for their bacterial contents using colony forming unit (CFU) determination (24, 25). Data indicated that tobramycin treatment significantly reduced *P. aeruginosa* infection level by ~1.3 log orders, highlighting tobramycin’s effectiveness in immunocompetent C57B mice (**Figure 1a**). We next assessed the impact of tobramycin and infection on IL-1β and TNF-α – (two key proinflammatory cytokines induced in response to *P. aeruginosa* infection (29, 38) - by ELISA. As expected, tobramycin alone did not affect the expression of these proinflammatory cytokines in the absence of infection, while infection without tobramycin led to modest increased levels of both cytokines as expected (**Figures 1b, c**). Notably, mice that were both infected and treated with tobramycin exhibited a significantly greater increase in IL-1β and TNF-α expression compared to those infected alone (**Figures 1b, c**). In line with these findings, expression levels of toll-like receptors (TLRs 1, 2, and 4) - key mediators of proinflammatory signaling and critical components for recognizing and mounting immune responses against extracellular pathogens including *P. aeruginosa* (35, 39, 40) - were also significantly upregulated in tobramycin-treated and infected wounds (**Figures 1d–f**). Further supporting these findings, histological analysis of inflammatory leukocytes using Hematoxylin and Eosin (H&E) staining, along with quantification of neutrophil content via myeloperoxidase (MPO) assessment by ELISA (15, 30), indicated significantly higher levels of inflammatory leukocytes and neutrophils in tobramycin-treated and infected wounds compared to infected wounds without tobramycin treatment (**Figures 1h, i**). Collectively, these data indicate that tobramycin boosts inflammatory responses toward infection in immunocompetent C57BL/6 mice.

### Tobramycin treatment fails to boost immunity against *P. aeruginosa* infection in immunocompromised NOD-scid IL2Rγ^null (NSG) mice

We next evaluated the potential synergy between tobramycin treatment and infection in NSG mouse, which is among the most immunocompromised mouse models. These mice lack mature T and B lymphocytes due to a *scid* mutation, resulting in nonfunctional T-cell and B-cell receptors (TCR and BCR) (41). Additionally, they lack functional natural killer (NK) cells due to impaired IL-15 signaling, which is essential for NK cell development and activity (41). NSG mice also exhibit compromised macrophage and dendritic cell function, leading to defects in antigen presentation, phagocytosis, and the secretion of key inflammatory cytokines (42, 43). Furthermore, neutrophils in NSG mice display impaired bactericidal functions, such as reduced phagocytic activity, decreased reactive oxygen species (ROS) production, and diminished chemotaxis (44, 45).

**Figure 2:**
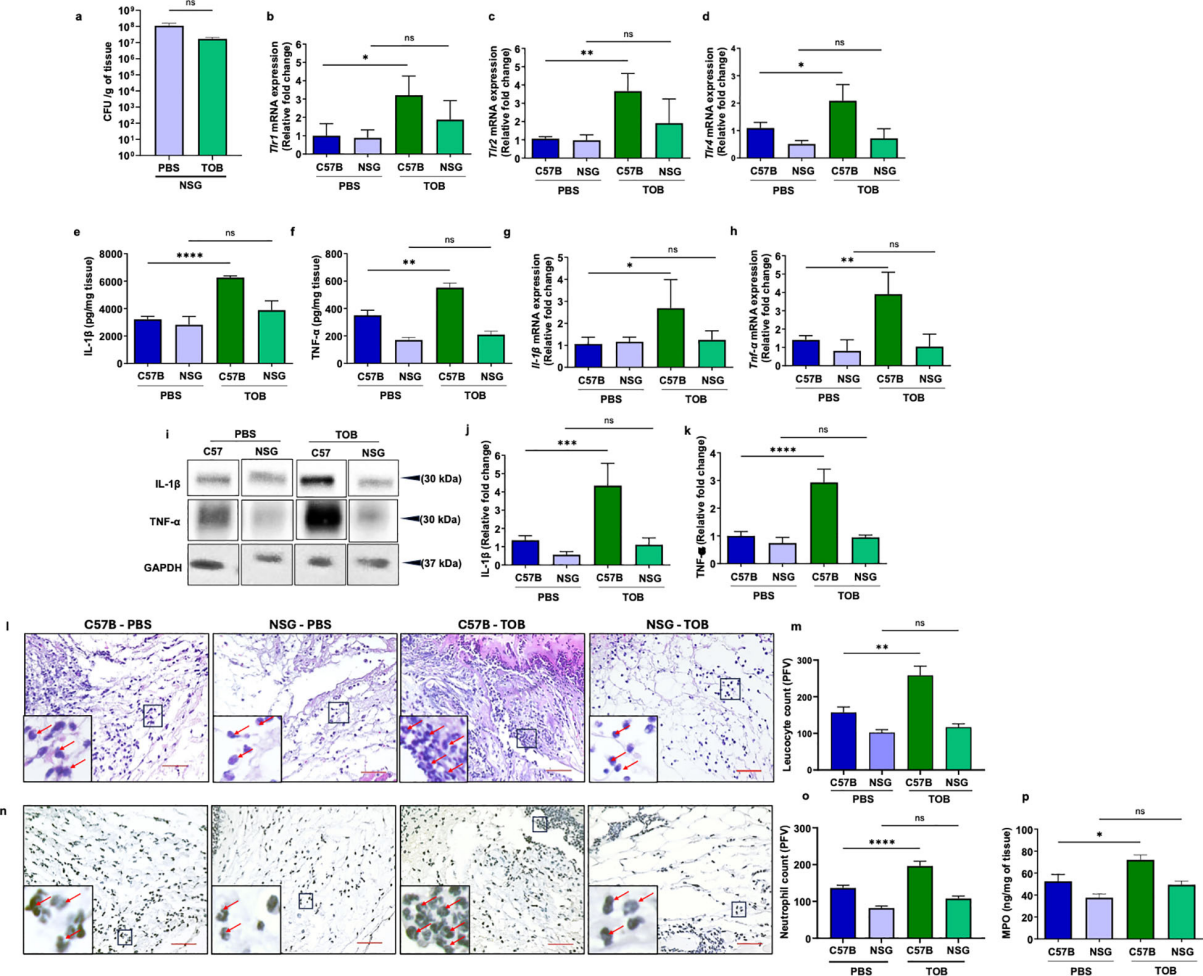
Tobramycin treatment fails to boost immunity against *P. aeruginosa* infection in NSG wounds. Mice were injected intraperitoneally with tobramycin (TOB) (3.5 mg/kg) or PBS (mock) 1h prior to wounding. Wounds from C57B and NSG mice were infected with PA103 (10^6^ CFU/wound). The wounds were collected after 24h and assessed for: **(a)** bacterial content by CFU count; **(b-d)** mRNA expression of Toll-like receptors (TLRs): *Tlr1, Tlr2* and *Tlr4* by RT-PCR (normalized to 18S); *(e-h);* proinflammatory cytokines IL-1β and TNF-α levels by ELISA **(e, f)** and by m-RNA transcription analysis by RT-PCR **(g-h)**; Western blot analysis of proinflammatory cytokines and the corresponding densitometer tabulated data for cytokines **(i-k)** IL-1β and k) TNF-α are shown as the relative fold change. Leukocytes contents assessed by histological analysis using H&E staining **(l, m)**, activated neutrophils contents by immunohistochemistry using anti-Ly6G antibody **(n, o)** and MPO ELISA **(p)**. Red scale bars = 50µm. Corresponding data were plotted as the Mean ± SEM. (N = 4). Statistical comparison between groups was determined using one-way ANOVA with Tukey’s *post hoc* test (ns= not significant, *p<0.05, **p<0.01; ***p<0.001).

The data indicated that, although tobramycin treatment showed a downward trend in infection levels in NSG mice, the reduction was not statistically significant, highlighting the important role of the immune system in its efficacy (**Figure 2a**). Supporting this observation, the tobramycin-induced enhancement of proinflammatory mediators—TLR1, TLR2, and TLR4 (assessed by RT-PCR); IL-1β and TNF-α (assessed by ELISA, RT-PCR, and Western blot); inflammatory leukocyte infiltration (assessed by H&E staining); and activated neutrophils (assessed by MPO ELISA)—was absent in PA103-infected NSG wounds (**Figures 2b–p**). Tobramycin has been shown to reduce neutrophil migration (46). We assessed the effects of tobramycin on chemotaxis in neutrophils isolated from both C57BL/6 and NSG mice (Materials & Methods) to determine if NSG neutrophils are more profoundly affected by tobramycin treatment. Our data confirmed that tobramycin reduced neutrophil chemotaxis, but this reduction was similar in both C57BL/6 and NSG neutrophils (**Supplementary Figure S1**), ruling out the possibility of that tobramycin worsens neutrophil dysfunction specifically in NSG mice. These data strongly suggest that the synergistic interaction between tobramycin and the host immune responses is disrupted in immunocompromised NSG mice, contributing to reduced antibiotic efficacy against infection. However, it remained unclear what is the reason for this synergy between tobramycin and immune responses in the context of infection.

### Elevated bioactive PAMPs drive the synergy between tobramycin and immune responses in immunocompetent mice

Driven by these data, we hypothesized that in immunocompetent mice, tobramycin-mediated bacterial killing increases the levels of bioactive (bioavailable) pathogen-associated molecular patterns (PAMPs). These bioactive PAMPs, in turn, enhance inflammatory responses by activating pattern recognition receptors (PRRs), such as Toll-like receptors (TLRs), and inflammasomes, thereby leading to increased production of proinflammatory cytokines. We define “bioactive” PAMPs as soluble, free microbial components capable of engaging and activating PRRs and inflammasomes, as opposed to bacterial products that remain structurally embedded within intact and viable bacteria. For example, free lipopolysaccharide (LPS) is a significantly more potent activator of TLR4 than an LPS molecule still buried in the outer membrane of live Gram‐negative bacteria and therefore inaccessible for TLR4 activation. We further postulated that this PAMP-driven synergy is diminished in immunocompromised hosts, resulting in reduced proinflammatory responses and decreased antibiotic effectiveness.

**Figure 3:**
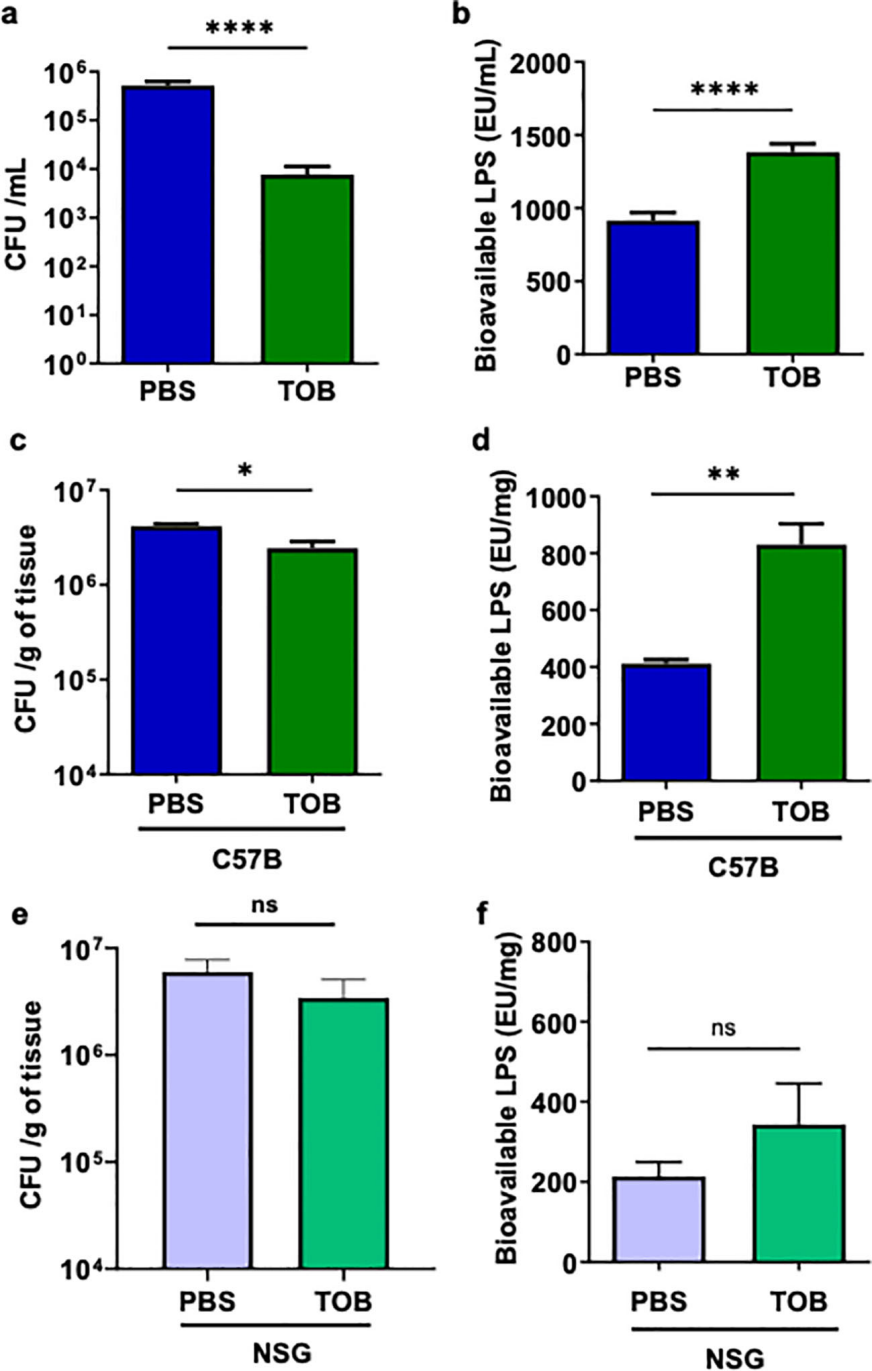
*P. aeruginosa* bacterial killing by Tobramycin increases bioactive (bioavailable) LPS levels in C57BL wounds, but not in NSG wounds. **(a, b)** PA103 bacteria cultured in LB (10^6^ bacteria/mL) were treated with Tobramycin (3.5 µg/mL) or PBS for 1 h Viable bacteria were quantified by CFU determination **(a)**, and bioactive LPS was measured using HEK-Blue hTLR4 reporter cells **(b)** (N = 6 replicates).**(c, d)** C57BL/6 and NSG mice received PBS or Tobramycin (3.5 mg/kg) by i.p. injection 1 h prior to wounding and infection with PA103 (10^6^ CFU/wound). Wound tissues collected after 1 h were analyzed for bacterial burden by CFU assay normalized to tissue weight **(c, e)** and for bioactive LPS using HEK-Blue reporter cells **(d, f)**. Data were normalized to wound tissue weight (N = 4 mice/group). Statistical comparisons were performed using one-way ANOVA with Tukey’s *post hoc* test (ns = not significant; *p < 0.05, **p < 0.01, ***p < 0.001). .

To test our hypothesis, we grew PA103 in liquid culture, then divided the culture in half, treating one half with tobramycin and the other with PBS for 1 hour. We then assessed the level of bioactive LPS—a key *P. aeruginosa* PAMP and the main TLR4 ligand (47)—using HEK-Blue hTLR4 reporter cells ( (29) and Methods). As expected, tobramycin significantly reduced the number of viable bacteria by nearly 1.5-log order (**Figure 3a**). Despite this reduction in viable bacteria and consistent with our hypothesis, the tobramycin-treated PA103 culture contained significantly higher levels of bioavailable LPS (**Figure 3b**). We next administered tobramycin by i.p. to immunocompetent C57B mice and challenged their wounds with PA103 infection. One hour after infection and treatment, we assessed bioactive LPS levels in the wound using HEK-Blue hTLR4 reporter cells. The results indicated that a 1-hour tobramycin treatment led to a modest (~0.3 log) but significant reduction in bacterial load (**Figure 3c**). However, despite this limited bacterial killing, tobramycin treatment significantly increased bioactive LPS levels in the wound (**Figure 3d**). In contrast, tobramycin treatment did not significantly reduce bacterial levels, although a downward trend was observed, nor did it significantly increase bioactive LPS levels in wounds, despite an upward trend in immunocompromised NSG mice (**Figures 3e, f**). The surprising reduced bacterial killing in NSG mice may be due to the important role of immune system in sensitizing bacteria toward antibiotics. For example, cationic antimicrobial peptides have been shown to sensitize bacteria toward antibiotic killing *in vivo* (48, 49). Combined, these data suggested that the lack of a tobramycin-induced boost in inflammatory responses toward infection in NSG mice may, at least in part, be due to reduced levels of PAMPs in these wounds.

### Topical treatment with bioactive PAMPs reduced infection in NSG mice by boosting inflammatory responses

**Figure 4:**
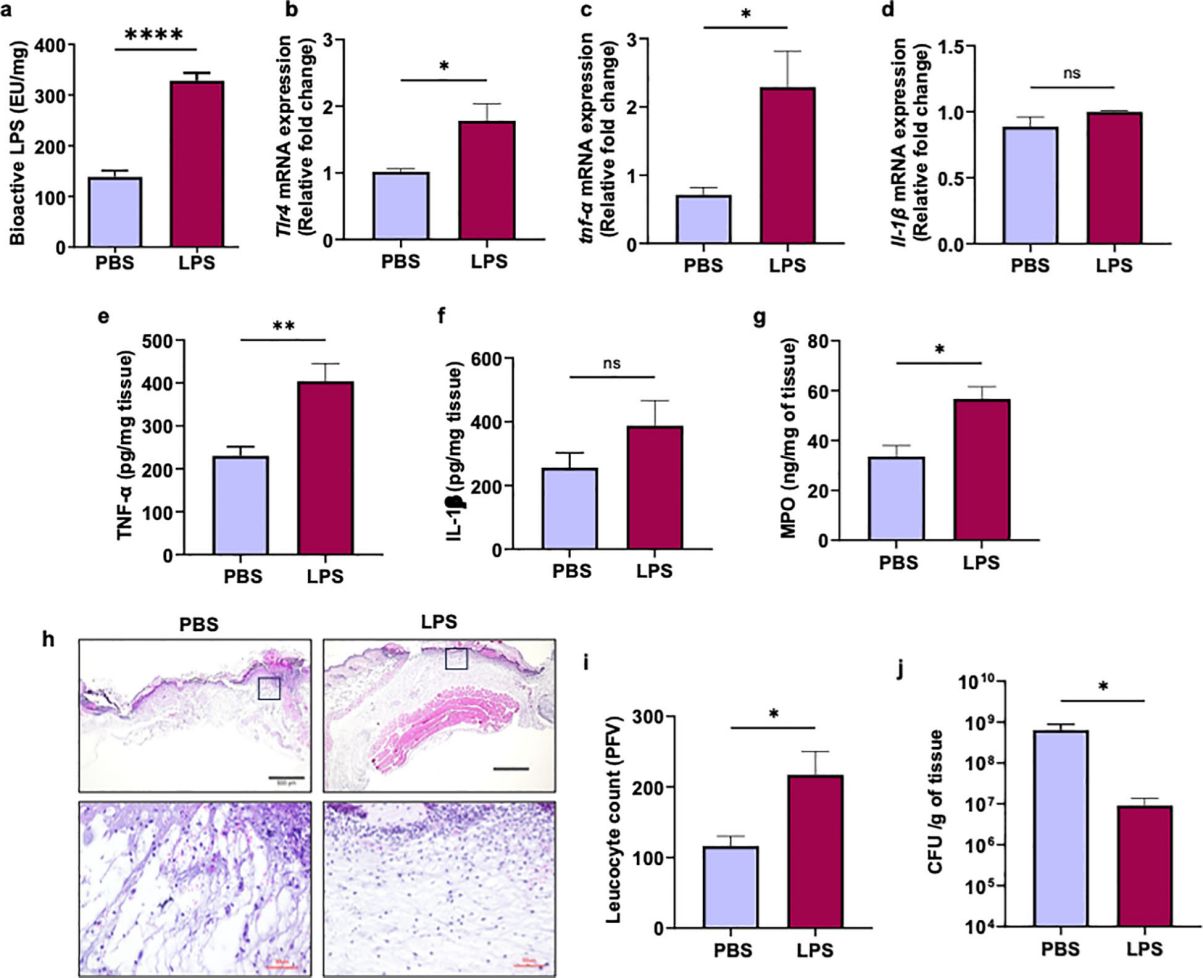
Topical LPS treatment boosts immunity against *P. aeruginosa* in NSG wounds. **(a–j)** Wounds in immunocompromised NSG mice were treated topically with PBS or lipopolysaccharide (LPS; 100 ng/wound) followed by infection with PA103 (10^6^ CFU/wound). One hour prior to wounding, mice received Tobramycin (3.5 mg/kg) by i.p. injection. **(a)** Wounds collected 24 h post-infection and analyzed for bioactive LPS using HEK-Blue reporter cells. **(b-j)** were examined for immune responses: mRNA expression of **(b)***Tlr4*, **(c)***Tnfα*, and **(d)***Il1β* by RT-PCR, confirmed by ELISA for **(e)** TNF-α and **(f)** IL-1β. **(g)** Activated neutrophil contents were assessed using MPO by ELISA. **(h–i)** Leukocyte infiltration was assessed by histological analysis (H&E staining). **(j)** Bacterial burden was determined by CFU determination. Black scale bars = 500µm and red scale bars = 50µm. (N = 4 mice/group). Statistical analysis was performed using one-way ANOVA with Tukey’s *post hoc* test (ns = not significant; *p < 0.05; **p < 0.01; ***p < 0.001; ****p < 0.0001).

To determine whether reduced levels of bioactive PAMPs in tobramycin-treated immunocompromised NSG mice contributed to their impaired ability to control infection, we topically applied a low dose of LPS to wounds in tobramycin-treated NSG mice, followed by PA103 infection challenge, and assessed the impact of LPS treatment on inflammatory responses and bacterial infection burden. As expected, LPS treatment significantly increased bioavailable LPS in NSG wounds (**Figure 4a**). Interestingly, LPS treatment significantly increased the expression of TLR4 and TNF-α, as measured by RT-PCR and ELISA (**Figures 4b, c, e**). Of note, IL-1β protein levels showed an upward trend by ELISA, although this increase did not reach statistical significance and was not observed at the mRNA level by RT-PCR (**Figures 4d, f**). LPS treatment also elevated leukocyte infiltration and neutrophil activation, as determined by H&E staining and MPO analysis, respectively (**Figures 4g–i**). Importantly, LPS topical application significantly reduced bacterial infection burden as assessed by CFU determination (**Figure 4j**).

To confirm that this effect was not specific to LPS, we repeated the experiment using another PAMP, *N*-formyl-methionyl-leucyl-phenylalanine (fMLP), applied topically at a low dose. Similar to LPS, fMLP treatment significantly increased bioavailable LPS, TNF-α levels, induced an upward trend in IL-1β although it did not reach statistical significance, stimulated leukocyte infiltration and neutrophil activation, and reduced bacterial burden by approximately 1-log order (**Supplementary Figures S2a–g**). Collectively, these data strongly suggest that insufficient PAMPs in tobramycin-treated, immunocompromised NSG mice contributes significantly to their reduced capacity to mount effective immune responses to control infection. Moreover, they demonstrate that exogenous PAMP application can at least partially restore innate immune responses and enhance infection control, even in a severely immunocompromised host like NSG mice.

### Tobramycin-induced boost in inflammatory responses toward infection is largely dependent on neutrophils

Host immune recognition of invading pathogens is highly redundant, as PAMPs can activate multiple PRRs, including membrane-bound TLRs and cytosolic sensors such as caspase-11 in mice and caspase-4 in humans, leading to proinflammatory cytokine production and recruitment of effector leukocytes (50–53). Neutrophils are the earliest and most essential responders to *P. aeruginosa* infection (54, 55). In addition to their antimicrobial functions, such as phagocytosis, production of reactive oxygen species (ROS), release of neutrophil extracellular traps (NETs), and antimicrobial peptides (AMPs) (56, 57), they also express various proinflammatory cytokines, such as TNF-α and IL-1β, which further amplify inflammatory responses against infection (58–62). Given this redundancy in PAMP sensing, we assessed the role of neutrophils in the tobramycin-induced enhancement of inflammatory responses in infected wounds. Additionally, antibiotics are reported to be less effective in neutropenic patients, including chemotherapy-treated cancer patients, even when pathogens remain antibiotic-sensitive (63, 64), providing another relevant immunocompromised model to test our hypothesis.

**Figure 5:**
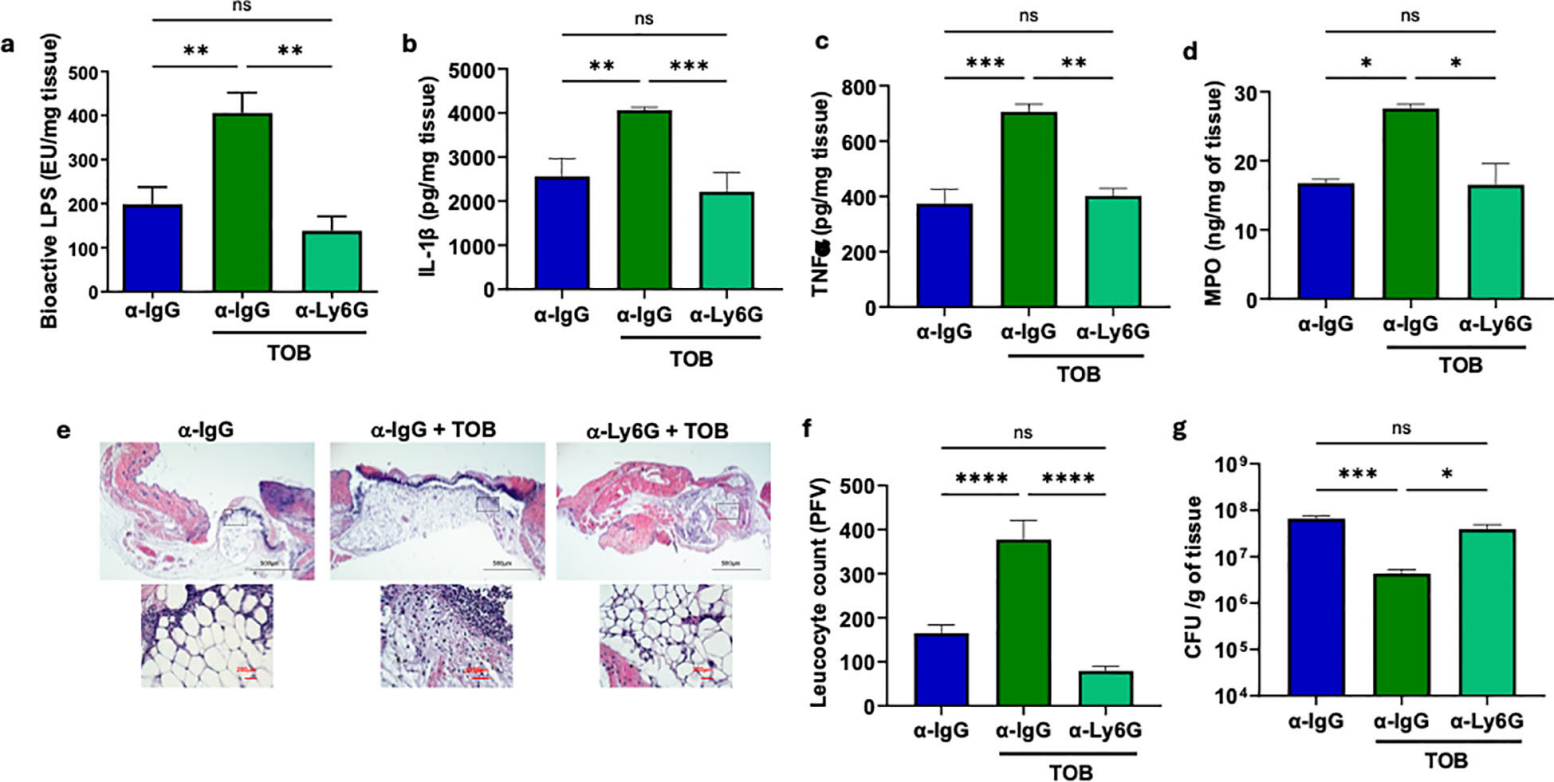
The synergy between Tobramycin and immune system is largely dependent on neutrophils. C57BL/6 mice were injected (i.p) with α-IgG and α-Ly6G prior to wounding as described in Materials & Methods, followed by infection with PA103 (10^6^ CFU/ wound). One hour prior to wounding, mice were treated with Tobramycin (3.5 mg/kg, IP). Wounds were collected 24 h after treatment and infection and assessed for: **(a)** bioactive LPS level using HEK-Blue hTLR4 reporter cells, Proinflammatory cytokine **(b)** IL-β, **(c)** TNF-α, and **(d)** neutrophil marker MPO by ELISA. **(e, f)** Leukocytes contents were assessed by histological analysis using H&E staining. **(g)** Bacterial burden in wounds was assessed by CFU determination. Black scale bars = 500µm and red scale bars = 50µm. Corresponding data were plotted as the Mean ± SEM. (N = 4 mice/group). Statistical analysis was performed using one-way ANOVA with Tukey’s *post hoc* test (ns = not significant; *p < 0.05; **p < 0.01; ***p < 0.001; ****p < 0.0001).

Toward this objective, we depleted the immunocompetent C57BL/6 mice of neutrophils by anti-Ly6G antibody injection ( (29, 30) and Materials & Methods) and assessed the impact of neutrophil depletion on inflammatory responses and infection in tobramycin-treated and infected C57BL/6 immunocompetent mice. Data indicated that neutrophil depletion abolished the tobramycin-induced increases in bioactive LPS, IL-1β, TNF-α, MPO, and leukocyte infiltration, while significantly increasing the infection burden (**Figures 5a–g**). Similarly, neutrophil depletion reduced bioactive LPS, IL-1β, TNF-α, MPO, and leukocyte infiltration, while significantly increasing the infection burden in LPS-treated wounds in NSG mice (**Supplementary Figures S4a–g**). Collectiveley, these data indicated that neutrophils are essential for mediating the tobramycin-induced boost in the inflammatory responses and improved infection control observed in tobramycin-treated immunocompetent C57BL/6 mice.

**Figure 6:**
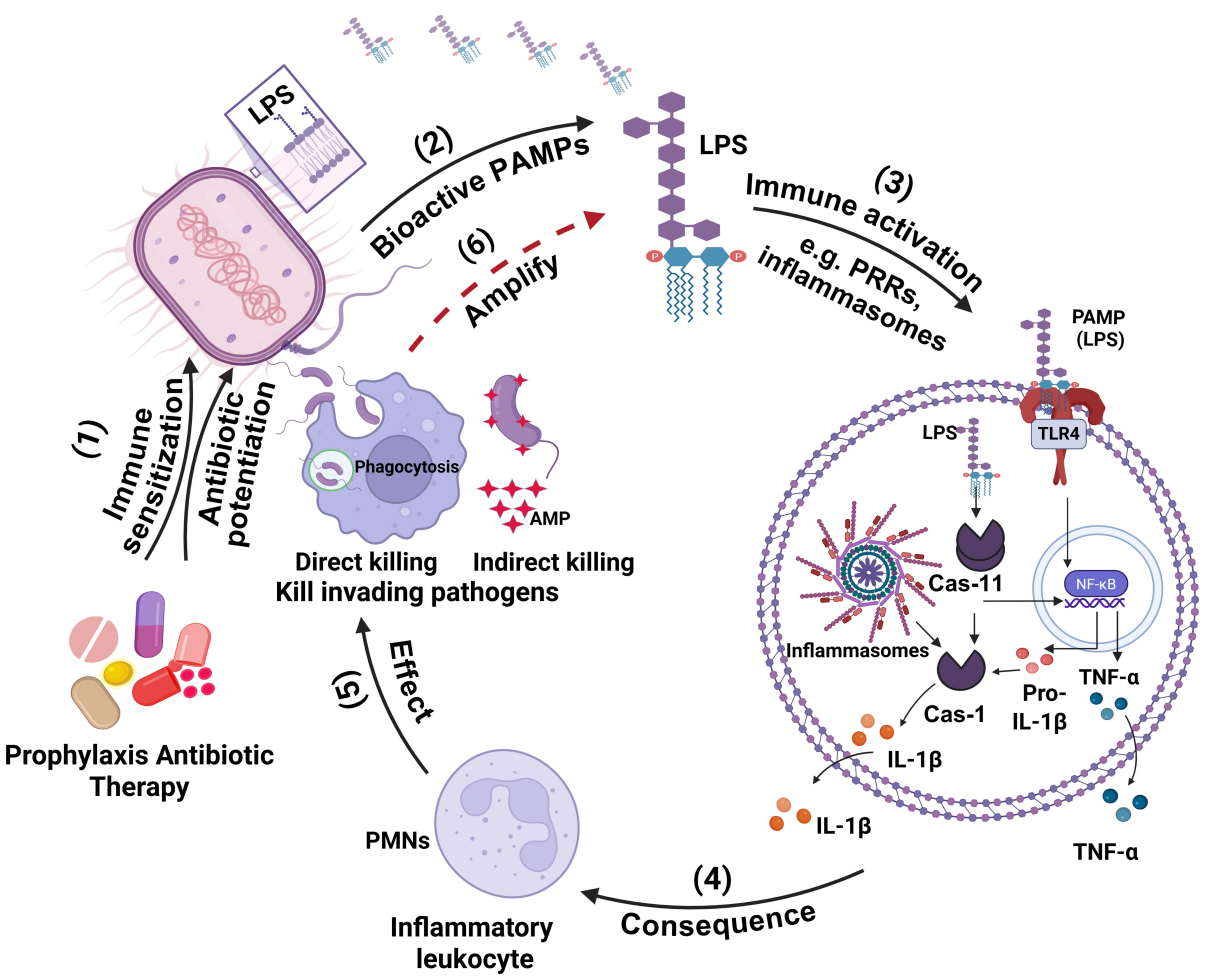
Schematic illustration of the model describing the synergistic interaction between antibiotics and the immune system. In this model, antibiotic-induced bacterial lysis is likely enhanced by the immune system sensitization, leading to the release of bioactive pathogen-associated molecular patterns (PAMPs) (1; Immune sensitization/Antibiotic potentiation). This results in elevated levels of bioavailable/bioactive PAMPs at the infection site (2, Bioactive PAMPs), which subsequently activate immune pathways, such as pattern recognition receptors (PRRs) and inflammasomes (3). Activation of these pathways increases the production of proinflammatory cytokines such as TNF-α and IL-1β at the infection site (3, PRRs & Inflammasomes activation). These cytokines facilitate the recruitment of inflammatory leukocytes, especially neutrophils, to the infected area (4; Consequence). The recruited neutrophils directly kill bacteria (5; Effect) while also amplifying inflammation through further cytokine release (6; Amplification). Together, this immune-mediated synergy enhances the *in vivo* effectiveness of antibiotics.

## Discussion

Our findings challenge the long-standing paradigm that antibiotics and host immunity operate as separate, sequential defenses against infection. Instead, we provide direct *in vivo* evidence that systemic antibiotic treatment can actively enhance host immune responses against infection, creating a synergistic feedback loop that improves infection control. In immunocompetent mice, tobramycin-mediated bacterial killing increased bioactive PAMPs, notably LPS, which in turn boosted proinflammatory cytokine production, and drove neutrophil recruitment and activation toward infection in wound. This immune amplification significantly improved infection control in immunocompetent mice. In contrast, this tobramycin-induced auxiliary boost in inflammatory responses did not occur in NSG mice rendering tobramycin ineffective in reducing infection in these immunocompromised mice. The data therefore position immune activation not as a passive beneficiary of antibiotic therapy but as a necessary partner for its maximal efficacy.

Clinically, reduced antibiotic effectiveness in immunocompromised patients has been well documented but this has been attributed largely to an inability to control residual bacterial populations after drug-mediated killing (3–5). Our data add a critical mechanistic layer to this understanding: the immune deficit is not only about inability to "mop up" remaining bacteria but also about missing an antibiotic-induced boost in inflammatory activation. This reframes the problem from being solely a matter of residual bacteria after antibiotic treatment to one of insufficient immune potentiation in immunocompromised host, due to reduced bioactive PAMPs.

Surprisingly, tobramycin neither reduced PA103 bacterial burden nor increased bioavailable LPS in NSG mice, raising the question as to why this occurred given the bacterium’s known sensitivity to tobramycin. Previous studies have shown that humoral immune components, such as cationic antimicrobial peptides (AMPs), can potentiate the bactericidal activity of antibiotics *in vivo* by sensitizing bacteria to antibiotic killing (48, 49). However, NSG mice have a severely impaired humoral immune branch (65). The absence of such synergy may blunt both antibiotic-induced bacterial lysis and PAMP release, leading to poor immune activation in NSG mice despite antibiotic treatment.

Importantly, topical administration of low level PAMPs (LPS and fMLP) was able to enhance signaling through pattern recognition receptors, increase the production of proinflammatory cytokines, and boost neutrophils trafficking and activation, resulting in substantial improvement in infection control in immunodeficient NSG mice. Considering that nearly all pathogens, including *P. aeruginosa*, have evolved multiple mechanisms to dampen inflammatory responses during infection (15, 66), our data suggest that bioactive PAMPs may have the theraputic potential to not only overcome some of pathogens’ anti-inflammatory virulence strategies but also to boost local inflammatory responses even in one of the most immunocompromised mice. These results have significant translational implications. In immunocompromised patients—such as those undergoing chemotherapy, organ transplantation, or living with chronic conditions like diabetes—systemic antibiotics alone may be insufficient because they fail to trigger the necessary innate immune amplification. Our data suggest that adjuvant therapies delivering controlled doses of PAMPs or PAMP mimetics may be able to overcome this deficit, boosting immune responsiveness and enhancing antibiotic effectiveness. Such strategies may be especially relevant in chronic wound care, where both bacterial burden and immune suppression contribute to poor healing outcomes, after surgical debridement to reset the wounds from chronic state into acute wounds (16, 24, 25, 29, 30, 35, 67, 68). It is important to emphasize that our findings do not recommend systemic administration of PAMPs. Rather, our data suggest that carefully controlled, low-dose topical application may help boost local innate immune responses against infection in settings where endogenous PAMP availability may be reduced, such as wounds. This localized approach minimizes the risk of systemic inflammatory responses while leveraging the beneficial immunomodulatory effects of PAMP signaling within the wound microenvironment.

In addition to reduced bioavailable LPS, it is likely that the impaired ability of NSG mice to control infection reflects broader defects in multiple immune pathways required for effective antibacterial responses. NSG mice lack functional T cells, B cells, and NK cells and exhibit deficiencies in neutrophil maturation and innate cytokine signaling, all of which limit their capacity to translate PAMP exposure into productive inflammatory activation (41–45). Thus, even when PAMP availability is experimentally restored, NSG mice may still fail to fully engage antimicrobial effector mechanisms that operate downstream of innate sensing. This impaired ability to amplify inflammatory signals likely reduces both direct immune-mediated bacterial killing and the immune-dependent sensitization of bacteria to antibiotic activity. Together, these immune defects provide a mechanistic explanation for diminished infection control in NSG mice that is independent of absolute LPS levels and further highlight the importance of host immune competence in mediating antibiotic efficacy *in vivo*.

Our neutrophil depletion experiments in mice indicate that these cells are not just innate immune effector cells but essential mediators of the antibiotic-induced immune boost, producing cytokines, amplifying inflammation, while also the primary cells responsible for combatting *P. aeruginosa*. Importantly, in NSG mice, topical treatment with exogenous PAMPs (LPS or fMLP) restored many of these immune functions, reducing bacterial burden in NSG mice and this was also largely dependent on neutrophil function. This demonstrates that immune potentiation strategies can work even in profoundly immunodeficient hosts, provided innate sensing pathways remain at least partially functional.

The schematic illustration of the model describing the synergistic interaction between antibiotics and the immune system is depited in **Figure 6**. In this model, antibiotic-induced bacterial lysis is likely enhanced by the immune system sensitization, leading to the release of bioactive pathogen-associated molecular patterns (PAMPs) (1; Immune sensitization/Antibiotic potentiation). This results in elevated levels of bioavailable/bioactive PAMPs at the infection site (2, Bioactive PAMPs), which subsequently activate immune pathways, such as pattern recognition receptors (PRRs) and inflammasomes (3). Activation of these pathways increases the production of proinflammatory cytokines such as TNF-α and IL-1β at the infection site (3, PRRs & Inflammasomes activation). These cytokines facilitate the recruitment of inflammatory leukocytes, especially neutrophils, to the infected area (4; Consequence). The recruited neutrophils directly kill bacteria (5; Effect) while also amplifying inflammation through further cytokine release (6; Amplification). Together, this immune-mediated synergy enhances the *in vivo* effectiveness of antibiotics.

In summary, our study shifts the conceptual framework from viewing antibiotics purely as bacterial killers to recognizing them as potential immune activators—an effect contingent on host immune competence. By uncovering the mechanistic role of bioactive PAMP release and neutrophil-mediated amplification, we open the door to combination strategies that deliberately engage innate immunity to improve infection outcomes, particularly in vulnerable patient populations.

## Data Availability

The original contributions presented in the study are included in the article/**Supplementary Material**. Further inquiries can be directed to the corresponding author.
