# Supplementary material for: Synergy between immune system and antibiotics drives infection control in mice
Source: Front Immunol. 2026 Jan 21;16:1719808. doi: 10.3389/fimmu.2025.1719808 (PMC12868242; doi:10.3389/fimmu.2025.1719808)
Supplement: Supplementary file 1 [file DataSheet1.docx]

***TITLE:***

***Synergy Between Immune System and Antibiotics Drives Infection Control in Mice***

***AUTHORS:***

Rajalekshmy G. Padmakumari ^1^, Ruchi Roy ^2^, Foyez Mahmud ^3^, Deepa Dehari ^1^, Getnet Tesfaw ^1^, Christi Thomas ^1^, Athena M. Soulika ^1,4^, Roslyn Rivkah Isseroff ^1,5^ , and Sasha H. Shafikhani ^1,6,7,8*^

**AFFILIATIONS:**

^1^ Department of Dermatology, University of California Davis, Sacramento, California, USA.

^2^ UICentre for Drug Discovery, College of Pharmacy, University of Illinois at Chicago, Chicago, Illinois, USA.

^3^ VeriSim Life Inc. San Francisco, CA, USA.

^4^  Department of Dermatology, Shriners Hospital for Children, Northern California, Sacramento, California, USA.

^5^ Dermatology Section, VA Northern California Health Care System, Mather, California, USA.

^6^ Microbiology Graduate Group (MGG), University of California Davis, Sacramento, California, USA.

^7^ Graduate Group in Immunology (GGI), University of California Davis, Sacramento, California, USA.

^8^ Cancer Center, University of California Davis, Sacramento, California, USA.

***CORRESPONDING AUTHOR**:

Sasha Shafikhani, Department of Dermatology, University of California Davis School of Medicine, Sacramento, CA, 95817, USA.

Email: [sshafikhani@health.ucdavis.edu](mailto:sshafikhani@health.ucdavis.edu)

**FIGURE S1**: **Impact of tobramycin on chemotaxis in C57BL/6 and NSG neutrophils**. Neutrophils were isolated from bone marrow of C57BL/6 mice and NSG mice and treated with tobramycin (0.035mg/mL) alone or a combination of tobramycin (0.035mg/mL) and LPS (100ng/mL). After 1 hour exposure, chemotaxis response toward fMLP (100nM) chemoattractant was assessed and the expressed as Relative Fluorescence Unit (RFU. The Corresponding data were plotted as the Mean ± SEM. (N=6/group). Statistical comparison between groups was determined using one-way ANOVA with Tukey’s post hoc test (ns= not significant, *p<0.05, **p<0.01, ***p<0.001, ****p<0.0001).

**FIGURE S2: Topical treatment with fMLP boosts immunity against *P. aeruginosa* in NSG wounds.** *(a–f)* Wounds in NSG mice were treated topically with PBS and fMLP (50ng/wound) followed by infection with PA103 (10^6^ CFU/ wound). One hour prior to wounding, mice also received Tobramycin (3.5 mg/kg) by i.p. injection. Wounds were collected 24 h post-infection and examined *(a)* bioactive LPS using HEK-Blue reporter cells; for pro-inflammatory cytokines: *(b)* TNF-α, *(c)* IL-1β by ELISA; *(d-e)* for leukocyte infiltration as assessed by H&E staining and histological analysis; *(f)* for activated neutrophil content by MPO analysis using ELISA; and *(g)* for their bacterial burden as determined by CFU determination. Black scale bars = 500µm and red scale bars = 50µm. (N=4 mice/group). Statistical analysis was performed using one-way ANOVA with Tukey’s post hoc test (ns = not significant; *p < 0.05; **p < 0.01).


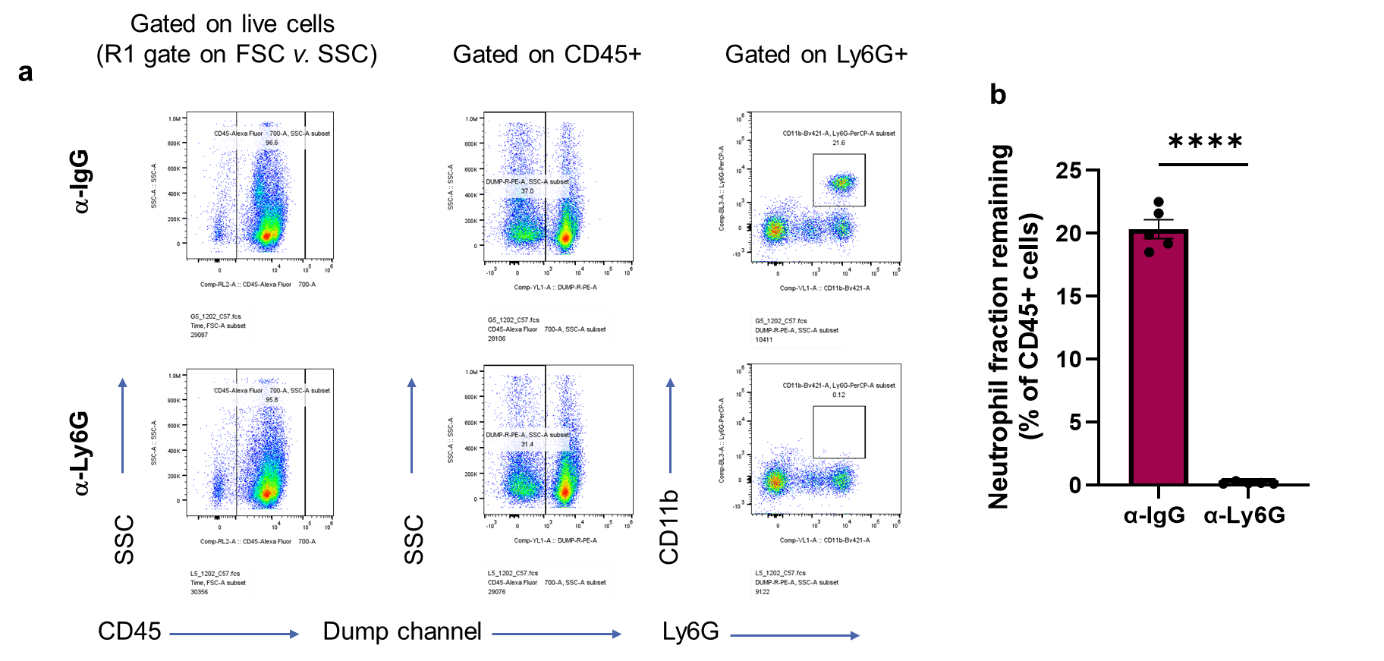


**FIGURE S3. Flow cytometry analysis for neutrophil depletion.** Neutrophil levels in the blood of C57BL/6 mice injected (i.p.) with either control IgG antibody (α-IgG) or anti-Ly6G antibody (a-Ly6G) were assessed by flow cytometry as described in the Materials and Methods. *(a)* Representative flow histograms are shown. For the gating strategy, live mononuclear cell populations were identified by gating on FSC-A versus SSC-A, CD45-positive immune cells staining versus SSC-A, and LIVE/DEAD staining versus SSC-A. The neutrophil population was then identified using CD11b versus Ly6G staining. *(b)* Tabulated data associated with neutrophil contents are shown. α-Ly6G reduced circulating neutrophils by 99.12% ± 0.42 compared to α-IgG control. Statistical analysis was performed by Student’s *t*-test. (N= 5 mice/group, **** p<0.0001).


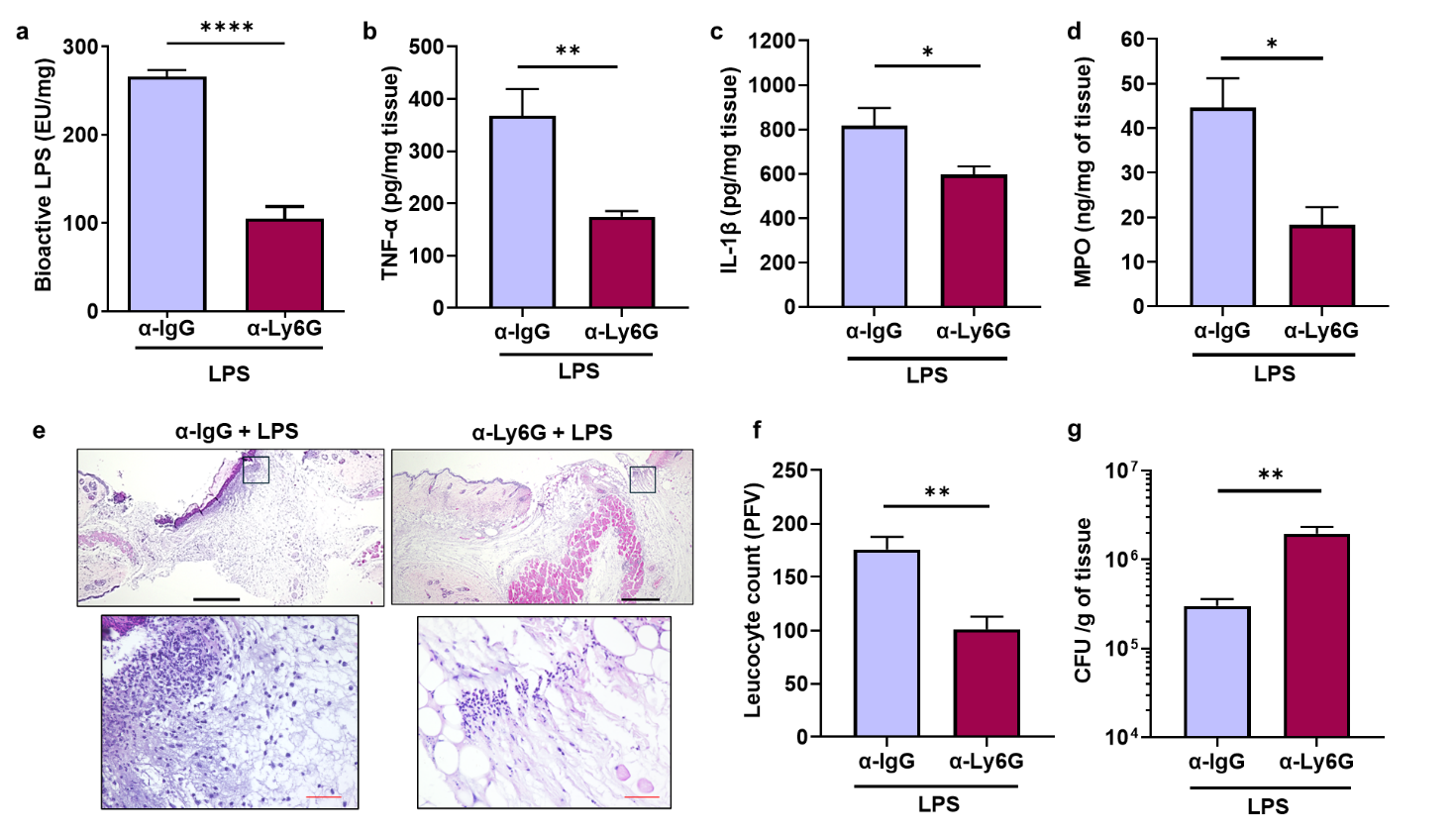


**FIGURE S4.** **The boost in the LPS-induced enhanced inflammatory responses and infection control in NSG mice is largely dependent on neutrophils**. NSG mice were injected (i.p) with α-IgG and α-Ly6G prior to wounding as described in Materials & Methods, followed by infection with PA103 (10^6^ CFU/ wound). One hour prior to wounding, mice were treated with Tobramycin (3.5 mg/kg, IP). Wounds were collected 24 h after treatment and infection and assessed for : *(a)* bioactive LPS using HEK-Blue reporter cells *(b)* TNF-α *(c)* IL-1β and *(d)* MPO were analyzed by ELISA. *(e, f)* Leukocytes contents assessed either by histological analysis using H&E staining and *(g)* bacterial content by CFU count. Corresponding data were plotted as the Mean ± SEM. Black scale bars = 500µm and red scale bars = 50µm. (N=4, ns= not significant, *p<0.05, **p<0.01).


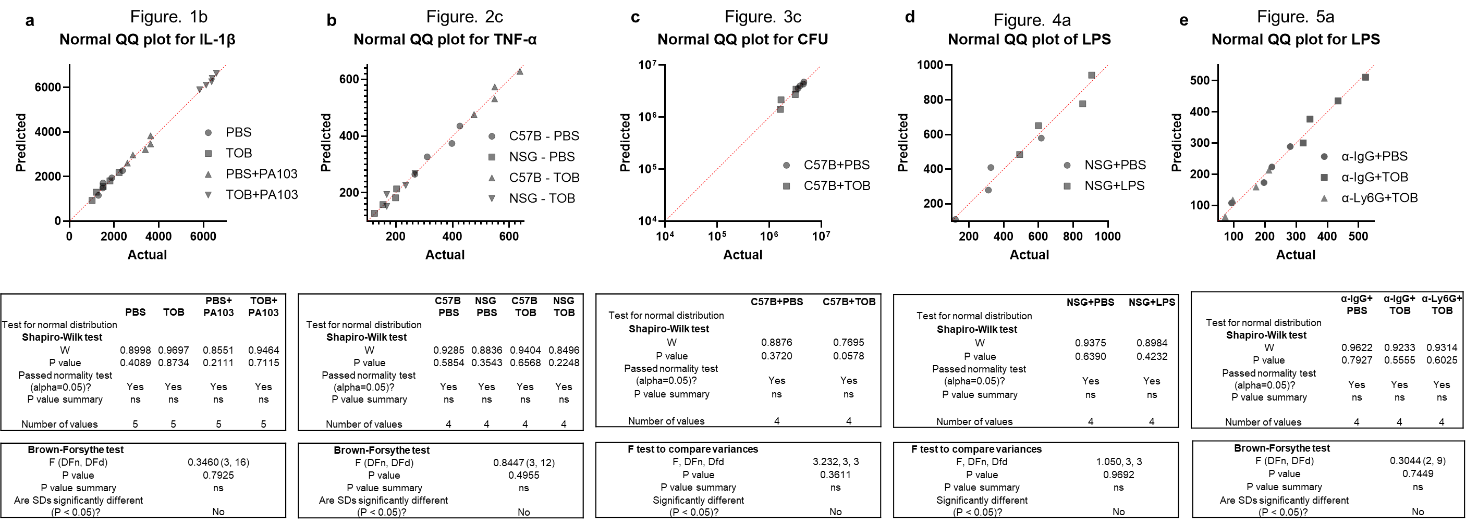


**FIGURE S5. Normality and homogeneity of variance assessments for datasets in Figures 1-5.** Graphical evaluations of normality and summary tables for homogeneity of variance are shown for representative datasets from each figure *(a–e)*. Normality was assessed using the Shapiro–Wilk test (appropriate for n < 30), and variance homogeneity was evaluated using the F-test for comparisons between two groups and the Brown–Forsythe test for comparisons involving more than two groups. All analyses were performed using GraphPad Prism (version 10.4.2). The assumptions of normality and equal variances were met prior to applying unpaired Student’s t-tests or one-way ANOVA, as appropriate. Panels include Q–Q plots for normality assessment and corresponding variance-test summary tables.


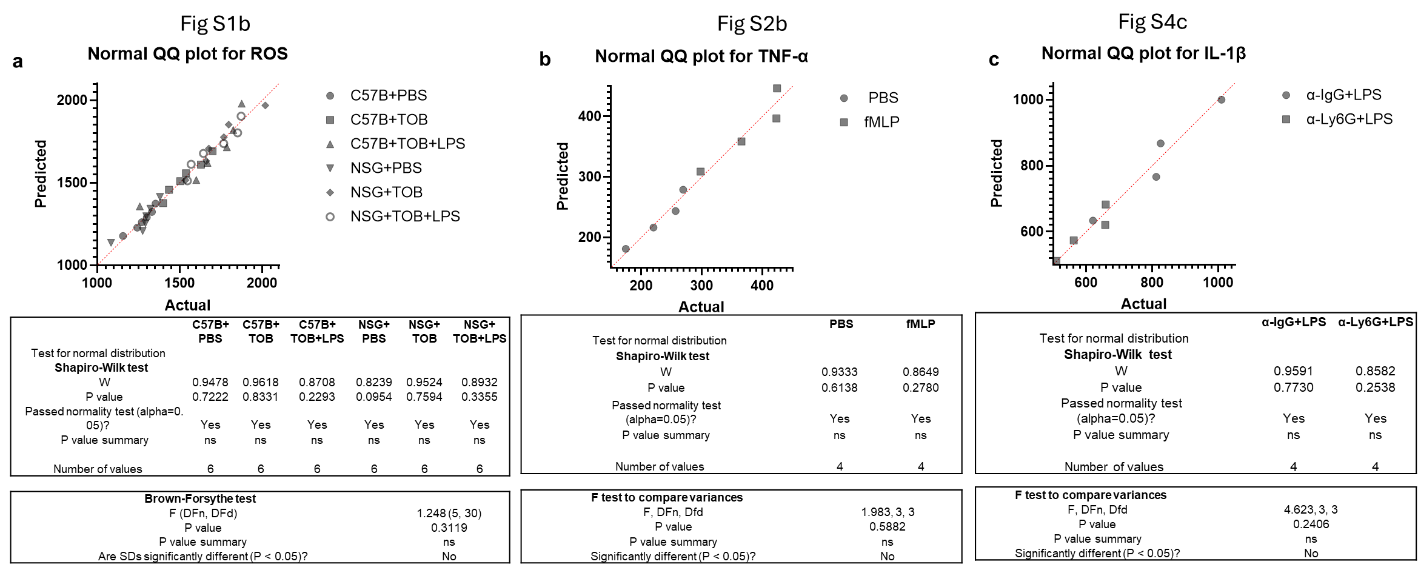


**FIGURE S6. Normality and homogeneity of variance assessments for datasets in Figures S1, S2, S4.** Graphical evaluations of normality and summary tables for homogeneity of variance are shown for representative datasets from each figure *(a–c).* Normality was assessed using the Shapiro–Wilk test (appropriate for n < 30), and variance homogeneity was evaluated using the F-test for comparisons between two groups and the Brown–Forsythe test for comparisons involving more than two groups. All analyses were performed using GraphPad Prism (version 10.4.2). The assumptions of normality and equal variances were met prior to applying unpaired Student’s t-tests or one-way ANOVA, as appropriate. Panels include Q–Q plots for normality assessment and corresponding variance-test summary tables.

**Supplementary Table 1. List of reagents and their sources.**

| Reagent type (species) or resource | | Designation | Source or reference | Identifiers | Additional information |
| --- | --- | --- | --- | --- | --- |
| Mouse strain background | | | | | |
| C57BL/6 J | C57BL/6 J | | Jackson laboratories | 000664 |  |
| NOD.Cg-Prkdcscid Il2rgtm1Wjl/SzJ | NSG | | Jackson laboratories | 005557 |  |
| Antibodies |  | |  |  |  |
| Antibody | Anti- Ly- 6G/Ly- 6C Monoclonal Antibody (RB6- 8C5) (Mouse monoclonal) | | Thermo Fisher Scientific | Cat. No. MA1- 10401 | For neutrophil depletion |
| Antibody | Anti- Mouse (G3A1) mAb IgG1 Isotype Control antibody (Mouse monoclonal) | | Cell Signaling Technologies | Cat. No. 5415 | For neutrophil depletion |
| Antibody | Alexa Fluor 700 anti-mouse CD45 | | BioLegend | Cat. No. 103128 | Flow cytometry |
| Antibody | Brilliant Violet 421™ anti-mouse/human CD11b | | BioLegend | Cat. No. 101251 | Flow cytometry |
| Antibody | PerCP/Cyanine5.5 anti-mouse Ly-6G Antibody | | BioLegend | Cat. No. 127616 | Flow cytometry |
| Antibody | PE anti-mouse CD3ε Antibody | | BioLegend | Cat. No. 152310 | Flow cytometry |
| Antibody | PE anti-mouse CD19 | | BioLegend | Cat. No. 115508 | Flow cytometry |
| Antibody | PE anti-mouse NK-1.1 | | BioLegend | Cat. No. 108707 | Flow cytometry |
| Antibody | TruStain FcX™ PLUS (anti-mouse CD16/32) | | BioLegend | Cat. No. 156604 | Flow cytometry |
| Antibody | Anti- Ly- 6G/Ly- 6C Monoclonal Antibody (RB6- 8C5) (Mouse monoclonal) | | Novus Biologicals | NBP2-00441 | Immuno histochemistry |
| Antibody | GAPDH Antibody Rabbit Polyclonal | | Proteintech | Cat. No. 1094-I-AP | Western blot |
| Antibody | Anti-TNF-α antibody | | Abcam | Cat. No. ab183218 | Western blot |
| Antibody | Anti-IL-1β antibody | | Abcam | Cat. No. ab254360 | Western blot |
| Antibody | Goat Anti-Rabbit IgG H&L (HRP) | |  | Cat. No. ab6721 | Western blot |
| ELISA |  | |  |  |  |
| Commercial assay or kit | Zombie NIR™ Fixable Viability Kit | | BioLegend | Cat. No. 423105 | Flow cytometry |
| Commercial assay or kit | HEK-Blue™ LPS Detection Kit assay | | InvivoGen | Cat. No. rep-lps2 | Detection and quantification of endotoxins (LPS) |
| Commercial assay or kit | TNF- α ELISA kit | | Thermo Fisher Scientific | Cat. No. 88- 7324- 88 | ELISA |
| Commercial assay or kit | IL- 1β ELISA kit | | Thermo Fisher Scientific | Cat. No. 88- 7013- 88 | ELISA |
| Commercial assay or kit | Myeloperoxidase (MPO) Mouse ELISA Kit | | Thermo Fisher Scientific | Cat. No. EMMPO | ELISA |
| Commercial assay or kit | SuperScript III First- Strand Synthesis System | | Thermo Fisher | Cat. No. 18080051 | RT-PCR |
| Commercial assay or kit | PowerUp™ SYBR™ Green Master Mix | | Thermo Fisher | Cat. No. A25742 | RT-PCR |
| Commercial assay or kit | EasySep™ Mouse Neutrophil Enrichment Kit | | STEMCELL Technologies | Cat. No. 19762 | Neutrophil isolation |
| Commercial assay or kit | ROS/Superoxide Detection Assay Kit (Cell-based) | | Abcam | Cat. No. AB139476 | ROS Detection |
| Reagents |  | |  |  |  |
| N- formyl- Met- Leu- Phe | fMLP (Synthetic peptide) | | Sigma | Cat. No. 59880- 97- 6 |  |
| Lipopolysaccharides | LPS (Escherichia coli O111:B4) | | Sigma Aldrich | Cat. No. L3012 |  |
| Reagent | Hematoxylin | | Thermo Fisher Scientific | Cat. No. 7111 L |  |
| Reagents | Eosin Y | | Thermo Fisher Scientific | Cat. No. 7211 L |  |
| Reagents | Bluing Reagent | | Thermo Fisher Scientific | Cat. No. 7301 L |  |
| Other | Collagenase D | | Sigma | Cat. No. 9001- 12- 1 |  |
| Other | Tobramycin | | Sigma | Cat. No. T4014 |  |
| Other | Buprenorphine Hydrochloride Injection | | MWI Veterinary Supply Co. | Cat. No. 060969 |  |
| Other | Lipopolysaccharides (LPS) | | Sigma Aldrich | Cat. No. L3012 |  |
| Primers for RT-PCR | | | | | |
| Oligonucleotides | *Il1* Forward: GCACTACAGGCTCCGAGATGAAC;  Reverse: TTGTCGTTGCTTGGTTCTCCTTGT | | Integrated DNA Technologies | NA |  |
| Oligonucleotides | *Tnf* Forward: TTGTCTACTCCCAGGTTCTCT, Reverse: GAGGTTGACTTTCTCCTGGTATG | | Integrated DNA Technologies | NA |  |
| Oligonucleotides | *Tlr1* Forward: GGTAGCAAGAGAAGTGGTGGAG  Reverse: CGATGGTGACAGTCAGCAGAAC | | Integrated DNA Technologies | NA |  |
| Oligonucleotides | *Tlr2* Forward: ACAGCAAGGTCTTCCTGGTTCC  Reverse: GCTCCCTTACAGGCTGAGTTCT | | Integrated DNA Technologies | NA |  |
| Oligonucleotides | *Tlr4* Forward: AGCTTCTCCAATTTTTCAGAACTTC  Reverse: TGAGAGGTGGTGTAAGCCATGC | | Integrated DNA Technologies | NA |  |
| Oligonucleotides | *18s* Forward: CGGAAAATAGCCTTCGCCATCAC  Reverse: ATCACTCGCTCCACCTCATCCT | | Integrated DNA Technologies | NA |  |
| Oligonucleotides | *Gapdh* Forward:  TTGGGTTGTACATCCAAGCA,  Reverse: CAAGAAACAGGGGAGCTGAG | | Integrated DNA Technologies | NA |  |
| Cell line |  | |  |  |  |
| Cell line | HEK-Blue™-4 cells | | InvivoGen | Cat.No.  rep-lps2 |  |
| Software / Purpose | | | | | |
| Statistics | GraphPad Prism | | GraphPad Prism  (version 10.4.2) | https://graphpad.com/scientific software/prism/ |  |
| Diagram | BioRender | | BioRender | <https://www.biorender.com/> |  |
| FIGURE 6 Schematic illustration of the model describing the synergistic interaction between antibiotics and the immune system.  *Created in BioRender. DEHARI, D. (2026)* [*https://BioRender.com/9xtse84*](https://BioRender.com/9xtse84)  *Agreement number: DQ297D38JK* | | | | | |

sss
